# Temperature-Controlled Switchable Photonic Nanojet Generated by Truncated Cylindrical Structure

**DOI:** 10.3390/ma16227209

**Published:** 2023-11-17

**Authors:** Ning Su, Weiming Zhang, Xintao Zeng, Pinghui Wu, Lina Cui, Xiaohui Chen

**Affiliations:** 1 Key Laboratory of Information Functional Material for Fujian Higher Education, Quanzhou Normal University, Quanzhou 362000, China; suning2022@163.com (N.S.); zwm08540854@163.com (W.Z.); zeng_xintao@163.com (X.Z.); 2 College of Textiles and Apparel, Quanzhou Normal University, Quanzhou 362000, China; 3 Research Center for Photonics Technology, Quanzhou Normal University, Quanzhou 362000, China; 18162886898@163.com

**Keywords:** photonic nanojet (PNJ), vanadium dioxide (VO_2_), finite-difference time-domain (FDTD)

## Abstract

We propose a novel micro-nano structure that can realize a photonic nanojet (PNJ) switch by adjusting the temperature, which is composed of a truncated cylinder coated with a thin vanadium dioxide (VO_2_) film. The influence of temperature on the maximum strength, full width at half maximum (FWHM), working distance, and focal length of the PNJ were studied by finite-difference time-domain (FDTD) method. The results demonstrate that the structure can adjust the open and close state of the PNJ by changing the temperature. A PNJ with varying characteristics can be obtained at both high and low temperatures, and the maximum intensity ratio of the PNJ can reach up to 7.25. This discovery provides a new way of optical manipulation, sensing and detection, microscopy imaging, optoelectronic devices, and other fields.

## 1. Introduction

Photonic nanojets (PNJs) refer to the highly focused, subwavelength light beams that are formed when light interacts with micro-scale or nanoscale dielectric structures or particles [1,2,3,4,5,6,7]. These unique optical phenomena arise from the scattering and interference effects occurring at the surface of objects. When light passes through these tiny structures, it undergoes multiple reflections, refractions, and interferences, resulting in the formation of concentrated, jet-like beams [8,9,10,11]. PNJs exhibit several remarkable characteristics. Firstly, they enable super-resolution imaging by achieving subwavelength focusing, exceeding the diffraction limit of conventional optics. Secondly, the intensity of light within PNJs is significantly enhanced, making them useful for applications requiring high optical power concentration. Thirdly, the focal spot size and position of the PNJs can be precisely controlled by manipulating the geometrical parameters and refractive indices of the structures involved [12,13,14,15,16]. These distinctive properties of PNJs have led to a broad range of potential applications. In the field of biophotonics, PNJs improve the imaging resolution of biological samples, enabling detailed observation and analysis of cellular structures. Chen et al. proposed a method for capturing and driving cells using high-quality PNJs generated by a specific micro-tapered fiber tip and made theoretical predictions, which is of great significance for the development of biophotonics [17]. PNJs can also be used for optical trapping and manipulation of small particles or molecules. Liu et al. designed a novel optical tweezer by integrating a fully dielectric hypersurface at the end of a dual-core optical fiber, which is extremely powerful for capturing small particles [18]. In addition, PNJs have an impact on lithography, and they can improve the accuracy and resolution of the lithographic process. Zhu et al. controlled the angle of incidence of the illumination to localize the photon jets within the cell defined by the microspheres, which enabled lithography of layered hypersurfaces [19].

PNJs possess unique characteristics that make them promising for various applications. However, a notable challenge in the field of PNJs is the difficulty in achieving efficient and controllable switching of their properties. Currently, altering the characteristics of PNJs often requires either changing the light source, modifying the structures involved, or adjusting the materials used [20,21,22,23,24,25,26,27]. These approaches may introduce additional complexity and limitations to the system. Consequently, there is a pressing need for novel strategies to overcome these challenges and enable more efficient and versatile switching of the PNJs’ properties.

In this study, we present a novel structure of truncated cylinders coated with a thin film of vanadium dioxide (VO_2_) at the incident end to control the properties of PNJ. By introducing this tailored structure, we exploit the unique phase transition properties of VO_2_ to achieve control over the PNJ’s characteristics. When the environmental temperature changes, the thin VO_2_ film undergoes a phase transition between its insulating state and the metallic state. This transition results in significant changes to the optical properties of the structure [28]. As a result, the PNJ formed at the output end exhibits distinct characteristics that can be controlled and modified. This structure provides a new way to efficiently switch and manipulate the PNJ’s characteristics without complex modifications or component replacement. In addition, it opens a way for the development of responsive and adaptive photonic devices.

## 2. Numerical Results and Discussion

### 2.1. The Model of Truncated Cylinder

**Figure 1:**
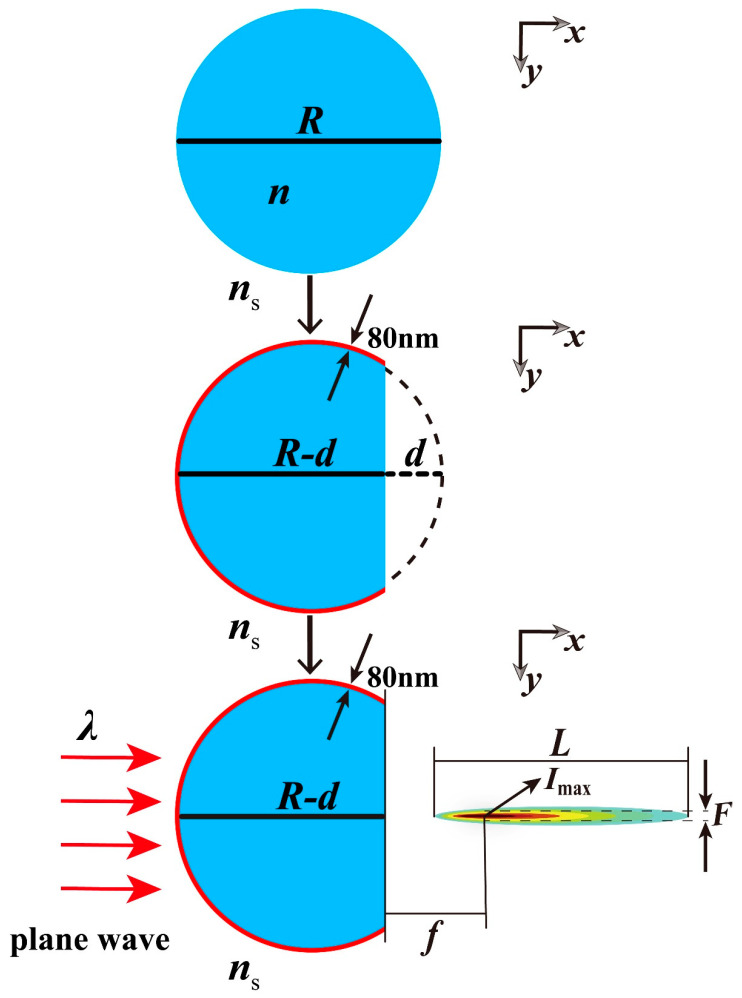
Schematic representation of the process of generating photonic nanojet from truncated cylindrical structures.

Figure 1 shows a schematic diagram of a truncated cylinder covered with a VO_2_ film to form a PNJ. A cylinder of infinite length in the height direction is truncated with the truncation direction parallel to the height direction and perpendicular to the diameter direction. The length of the diameter of this cylinder is *R*. After truncation, the diameter direction is divided into two parts of length *d* and length *R*–*d*, and the part of length *R*–*d* is retained as the truncated cylinder. Therefore, we define *d* as the truncated thickness. The untruncated surface of the truncated cylinder is uniformly covered with a VO_2_ film with a thickness of 100 nm, which together with the truncated cylinder forms the complete structure. The incident light is a plane wave with wavelength *λ*, incident along the *x*-axis. When the light interacts with the structure, a PNJ is generated behind the truncated surface. In Figure 1, *f* represents the focal length, which corresponds to the distance from the truncation plane to the point of maximum intensity in the PNJ. *F* denotes the full width at half maximum (FWHM), which represents the *y*-axis width corresponding to the PNJ attenuation from maximum intensity to half of the maximum intensity. According to *F*, we can get the focusing ability of PNJ. *L* is the effective length, which refers to the distance between two points along the *x*-axis when the light intensity decays to 1/e times the maximum light intensity. *I*_max_ is the maximum intensity.

### 2.2. The Simulation of Truncated Cylinder

The finite-difference time-domain (FDTD) method is a numerical technique used to solve the equations of motion for electromagnetic waves. It is widely employed in computational electromagnetics and photonics research. FDTD discretizes both space and time, allowing for the simulation of wave propagation and interaction effects in complex structures. In FDTD, the space being simulated is divided into a grid of discrete cells, and each cell represents a small volume within the structure. The electromagnetic field quantities, such as electric and magnetic fields, are calculated at each grid point in each time step. By solving Maxwell’s equations iteratively in time, the behavior of electromagnetic waves can be simulated accurately [29]. In this model, the simulation region was divided into cubes with a length of 10 nm, and perfect matching layers (PML) were used as the absorption boundary conditions. The diameter of the cylinder (*R*) was set to 10 μm, and the refractive index of the cylindrical material (*n*) was set to 1.55. The incident wave’s wavelength (*λ*) was chosen after iterative simulations and tests, and ultimately set to 450 nm. The plane wave propagated along the *x*-axis and exhibited linear polarization with the polarization direction in the *y*-axis. The surrounding medium was air.

**Figure 2:**
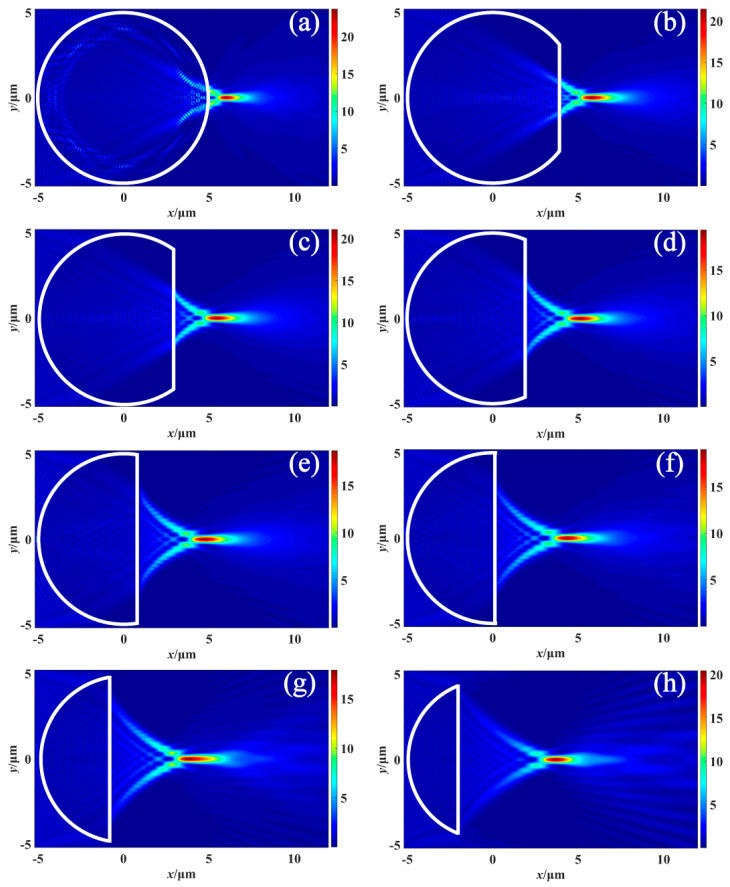
Intensity distribution of PNJ along the *y*-axis with uniformly increased thickness *d* of cylindrical cut (**a**) *d* = 0 μm (**b**) *d* = 1 μm (**c**) *d* = 2 μm (**d**) *d* = 3 μm (**e**) *d* = 4 μm (**f**) *d* = 5 μm (**g**) *d* = 6 μm (**h**) *d* = 7 μm.

Figure 2 illustrates changes in the intensity distribution of the light field in the *x*-*y* plane as the truncated thickness of the cylinder uniformly increases. From the sequence plots in Figure 2, it is evident that with the increase of *d*, the PNJ continuously moves away from the outgoing surface and its length increases considerably. Additionally, the distance (*f*) of the point with maximum light intensity from the outgoing surface also increases.

**Figure 3:**
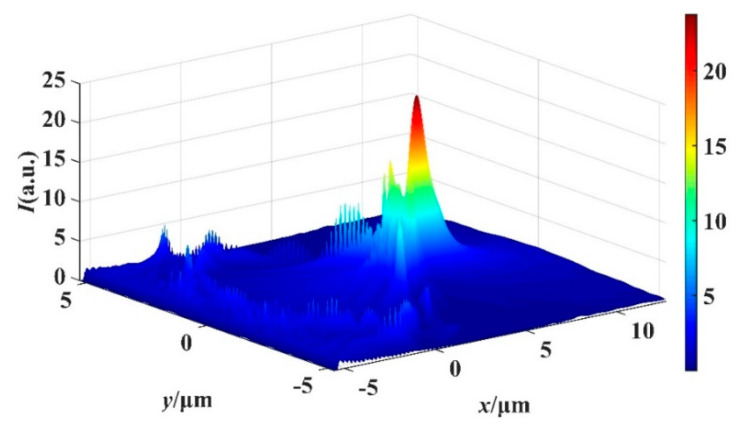
When *d* = 0 μm, three-dimensional light field distribution of the structure.

Figure 3 shows the three-dimensional intensity distribution of the structure when *d* = 0 μm. As can be seen from the figure, PNJ shows a high degree of symmetry. In addition, as the incident light propagates through the structure, the incident light undergoes interference and diffraction effects due to changes in the shape of the structure, which causes a localized focusing effect. In the *x*-axis direction, the incident light first experiences the focusing effect and reaches peak intensity. As the light continues to propagate, the focusing effect diminishes, resulting in a gradual decrease in light intensity.

**Figure 4:**
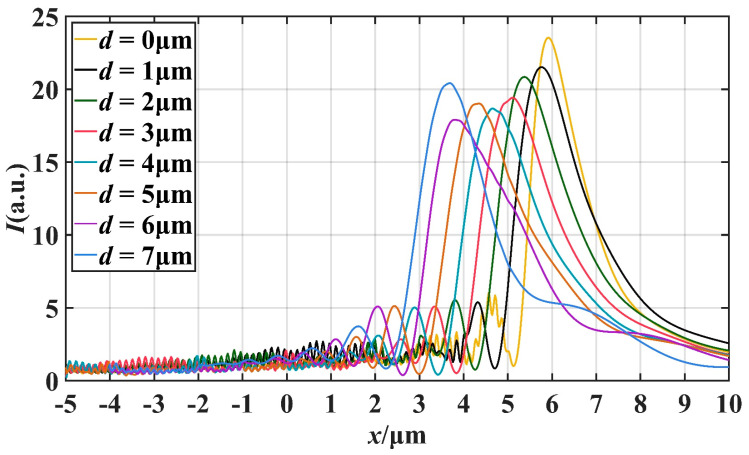
The influence of the truncation thickness *d* on the *x*-axis intensity distribution.

**Figure 5:**
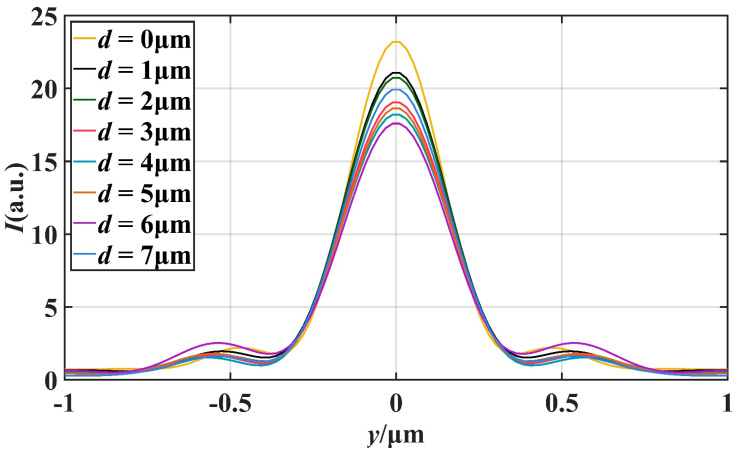
Comparison of FWHM under different truncation thickness *d*.

Figure 4 illustrates the variation of the light field intensity along the *x*-axis of the PNJ. By observing the curves in the figure, we can understand that the peak intensity of the PNJ is basically in a decreasing trend with an increase of the truncation thickness *d*, but a certain degree of increase occurs at *d* = 5 μm and *d* = 7 μm. When *d* increases, the maximum strength point of PNJ always moves to the negative direction of *x*-axis. In addition, when *d* = 7 μm, the value of *L* is the largest, reaching 2.15 μm, which is equivalent to 4.78 times of the wavelength. Figure 5 illustrates the distribution of the intensity of the light field along the longitudinal direction near the point of maximum light intensity at the PNJ. We can calculate the FWHM of the resulting PNJ at different *d* and determine the degree of focusing of the resulting PNJ based on the FWHM. It can be seen in Figure 5 that the FWHM reaches the minimum when *d* = 0 μm, and its value is 0.324 μm.

The quality factor is an important parameter used to assess the quality of a PNJ beam. It provides a quantitative measure of the focusing performance and localization of the PNJ beam. Specifically, the quality factor can be calculated using the following equation [30]:

(1) $$Q=\frac{I_{max}\times L}{F}$$

where *L* is effective length, *I*_max_ is the maximum intensity and *F* is FWHM. It is apparent from the equation that *I*_max_, *L*, and *F* all affect the magnitude of *Q*. Larger *I*_max_ and *L*, as well as smaller *F*, increase the value of *Q*, thus optimizing the quality of the PNJ. By combining the information obtained from Figure 4 and Figure 5, we can extract valuable data regarding the changes in the properties of the photonic nanojets as *d* increases. These data are summarized and presented in Table 1, providing a comprehensive overview of how variations in *d* affect key characteristics such as the *I*_max_, *L*, and *F*.

As shown in Table 1, the focal length *f* of the PNJ increases as the truncation thickness *d* decreases. When *d* = 7.0 μm, *f* reaches 6.70 μm, which is equivalent to 14.89 times the wavelength. A larger focal length allows for higher local intensities at greater distances, allowing light and matter to interact at greater distances. From *d* = 0 μm to *d* = 2.0 μm, the quality factor *Q* firstly increases and then decreases, and reaches an extreme value of 105.95 when *d* = 1.0 μm. From *d* = 2.0 μm to *d* = 7.0 μm, the quality factor *Q* obviously decreases at first, then increases continuously, and reaches the maximum value of 123.79 when *d* = 7.0 μm. From *d* = 0 μm to *d* = 2.0 μm, the effective length *L* first increases and then decreases. After that, *L* also increases with *d* increasing, and reaches a maximum of 2.15 μm at *d* = 7.0 μm, which is equivalent to 4.78 times the wavelength. *F* is the narrowest at *d* = 0 μm, only 0.72 times the wavelength. With the increase of *d*, the value of *F* does not change much, floating around 0.8 times the wavelength, which indicates that PNJ always maintains excellent focusing ability. At *d* = 0 μm, *d* = 1.0 μm, *d* = 2.0 μm, *d* = 7.0 μm, the maximum intensity *I*_max_ can reach more than 20, and at *d* = 0 μm, the maximum is 23.64.

### 2.3. Simulation of Truncated Cylinder-Covering VO_2_ Film

We compared the quality factor and focal length of the truncated cylinders to produce PNJ comprehensively and selected the cases of *d* = 1.0 μm and *d* = 7.0 μm. It is worth noting that the VO_2_ material is an excellent phase change material with a phase change temperature of 68 °C. Below the phase transition temperature, VO_2_ is in the insulating phase with insulating properties, and its lattice structure shows a large distortion and electron localization phenomenon, resulting in high resistivity and difficult free flow of charge. When plane waves are irradiated on the VO_2_ film, the energy of the light cannot be efficiently converted into electrical energy, resulting in difficult absorption. On the contrary, when the surrounding temperature rises above the phase transition temperature, the lattice structure of VO_2_ is reconstructed, resulting in more free movement of electrons, significantly reduced resistivity, and thus showing high conductivity, that is, from the insulating phase to the metallic phase. This phase transition is accompanied by a rearrangement of electrons and a change in the lattice, allowing VO_2_ to absorb light better [31].

**Figure 6:**
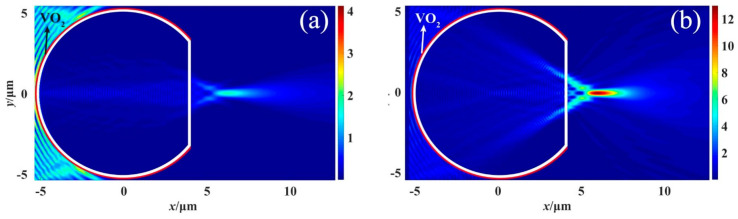
Intensity distribution in the *x*-*y* plane at different ambient temperatures when *d* = 1 μm: (**a**) *T* = 25 °C, (**b**) *T* = 85 °C.

**Figure 7:**
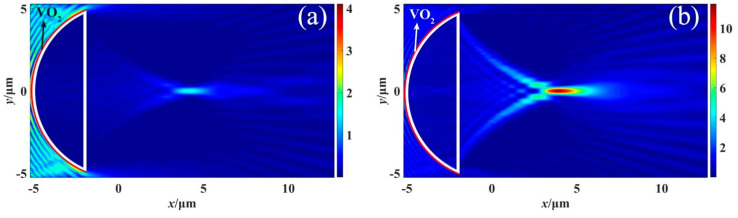
Intensity distribution in the *x*-*y* plane at different ambient temperatures when *d* = 7 μm: (**a**) *T* = 25 °C, (**b**) *T* = 85 °C.

To investigate the effect of temperature on the PNJ phenomenon, we conducted simulations under two different ambient temperature conditions: 25 °C and 85 °C. We maintained the same incident wave conditions as described previously. These simulations enabled us to observe and analyze the resulting PNJ phenomenon under different temperature scenarios. Figure 6 shows the *x*-*y* plane intensity distribution at different temperatures for *d* = 1 μm. And Figure 7 shows the *x*-*y* plane intensity distribution at different temperatures for *d* = 7 μm.

Figure 6 and Figure 7 clearly illustrate the distinctive differences in the PNJ phenomenon between the metallic and insulating phases of the structure. Under the metallic phase, the structure produces a distinct PNJ with significant strength distribution. Conversely, under the insulating phase, the structure absorbs the incident wave, resulting in an extremely weak jet observed only on the right side of the structure.

**Figure 8:**
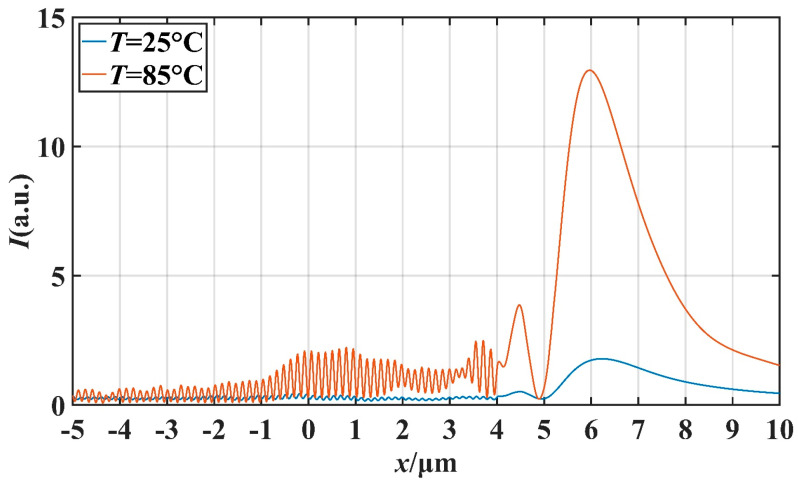
When *d* = 1 μm, the influence of *T* on the *x*-axis intensity distribution.

**Figure 9:**
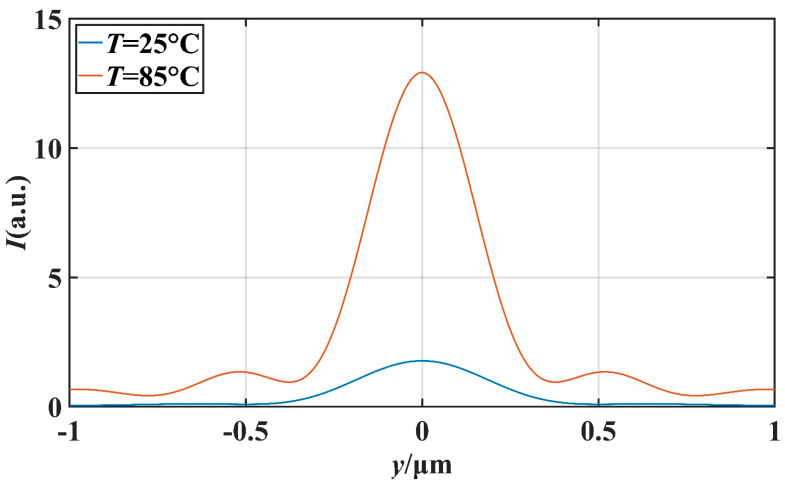
When *d* = 1 μm, FWHM comparison at different temperatures *T*.

**Figure 10:**
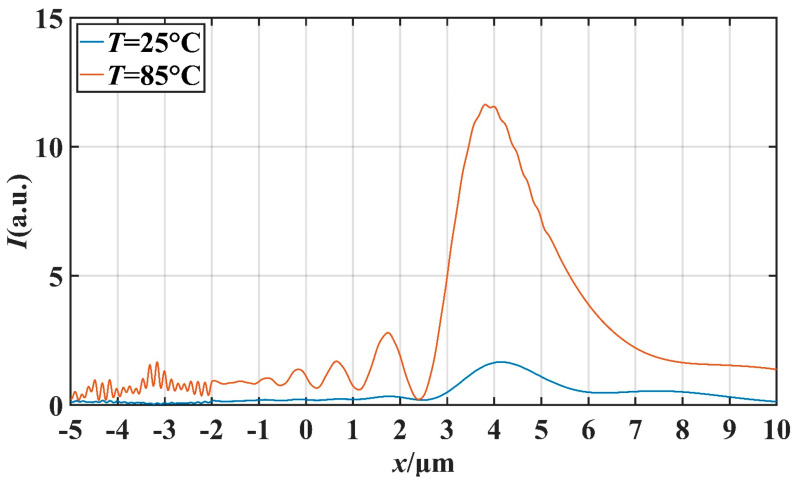
When *d* = 7 μm, the influence of *T* on the *x*-axis intensity distribution.

**Figure 11:**
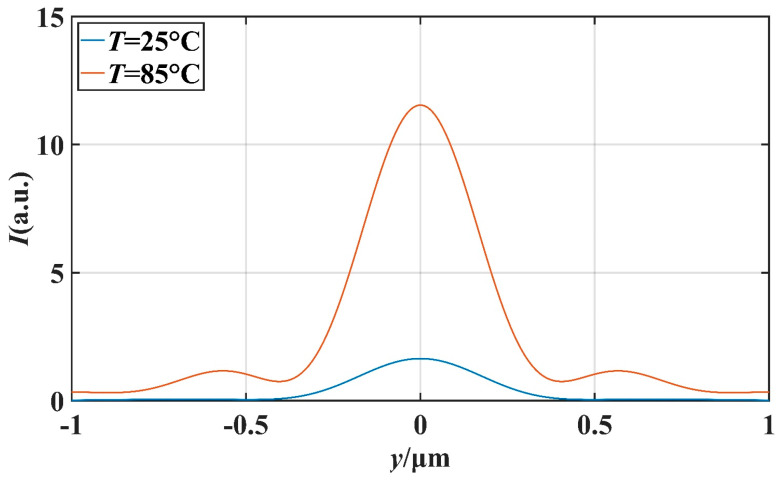
When *d* = 7 μm, FWHM comparison at different temperatures *T*.

In order to further investigate the potential application of this micro-nano structure in the field of optical switching, we studied the effect of temperature on the characteristics of the PNJ under different *d* values. We mainly studied the effect of temperature on light intensity on the *x*-axis and the effect of temperature on the FWHM. Setting the ambient temperature as *T*, we simulated at *T* = 25 °C and *T* = 85 °C. Figure 8 and Figure 9 describe the transverse and longitudinal intensity distribution of the PNJ when *d* = 1 μm. When *T* = 85 °C, the maximum intensity of the PNJ is about 13.98 times of the incident wave intensity. When *T* = 25 °C, the maximum intensity of the PNJ is only about 1.79 times of the incident wave intensity. Similarly, Figure 10 and Figure 11 describe the transverse and longitudinal intensity distribution of PNJ when *d* = 7 μm. When *T* = 85 °C, the maximum intensity of the PNJ is about 11.61 times of the incident wave intensity, while when *T* = 25 °C, the maximum intensity of the PNJ is only about 1.67 times of the incident wave intensity. We present statistics on the changes in the PNJ characteristics obtained at this time, as shown in Table 2 and Table 3.

It can be seen in the above table that when *d* = 1.0 μm, the quality of the PNJ is higher at high temperature, and the value of *Q* can reach 89.78. At low temperature, the value of *Q* is only 14.95, and the value of *Q* at high temperature reaches 6.01 times of the value of *Q* at low temperature. At high temperatures, the *I*_max_ of the PNJ is 12.98, while at low temperatures, the *I*_max_ of the PNJ is only 1.79, and the maximum strength at high temperatures is 7.25 times that at low temperatures. It is evident that the intensity of the PNJ undergoes a significant change. For *F*, *F* is 0.072 μm smaller at high temperatures than at low temperatures, which means that the PNJ at high temperatures is narrower and has higher spectral resolution. The effective length *L* at high temperature is reduced by 1.10 μm compared to low temperature, and the focal length *f* is also reduced by 0.23 μm. When *d* = 7.0 μm, the PNJ is also higher quality at high temperature, and the value of *Q* can reach 87.01. At low temperature, the value of *Q* is only 11.02, and at high temperature, the value of *Q* reaches 7.90 times that of the value of *Q* at low temperature, which is a more significant gap compared with the case of *d* = 1.0 μm. At high temperatures, the *I*_max_ of the PNJ is 11.61, while at low temperatures, the *I*_max_ of the PNJ is only 1.67, and the maximum strength at high temperatures is 6.95 times that at low temperatures. For *F*, *F* is 0.003 μm smaller at high temperature than at low temperature, and the change is not significant. The effective length *L* at high temperature increases by 0.33 μm compared with that at low temperature, and the focal length *f* also increases by 0.70 μm, which indicates that the high temperature causes the PNJ to be farther away from the structure and the working distance to be longer.

## 3. Conclusions

In this study, we showed that by adjusting the external conditions or structural parameters, the shape, position or intensity of PNJ can be reversibly changed, thus realizing the switching of PNJ characteristics. We propose a novel structure for PNJ generation: by changing the external temperature, the VO_2_ thin film can switch between the metal phase and the insulating phase, thus changing the absorption of the structure to the incident light and finally leading to changes in the PNJ characteristics formed at the exit end. The results show that the structure can adjust the open and close state of the PNJ by changing the temperature; the generated PNJ is high quality, which provides a new method for the development of optical switching.

It is worth noting that after undergoing a phase transition, VO_2_ requires a certain amount of time to transition from one state to another, and this transition time depends on the material’s prior phase transition history [32]. In addition, VO_2_ films may decompose under high temperature and high humidity. Thus, our study merely provides a theoretical basis. In future study, we will further consider the phase transition kinetics of the materials as well as the material formulation and preparation process of the VO_2_ films to evaluate their performance in practical applications more accurately.

All in all, this study achieves reversible switching of the PNJ’s properties through structural design and temperature control and demonstrates the potential for optical switching through material phase transitions. This study brings new possibilities for the optimal design of optical devices and systems and related applications in photonics and is of great significance for the further development of optical manipulation, sensing and detection, microscopy and imaging, and optoelectronic devices.

## Figures and Tables

**Table 1:** Characteristic parameters of PNJ generated by different *d*.

*d* (μm)	*I*_max_ (a.u.)	*F* (μm)	*L* (μm)	*Q*	*f* (μm)
0	23.64	0.324 (0.720*λ*)	1.29 (2.87*λ*)	94.12	0.92 (2.07*λ*)
1.0	21.56	0.350 (0.778*λ*)	1.72 (3.82*λ*)	105.95	1.76 (3.91*λ*)
2.0	20.89	0.352 (0.782*λ*)	1.70 (3.78*λ*)	100.89	2.37 (5.27*λ*)
3.0	19.47	0.360 (0.800*λ*)	1.71 (3.80*λ*)	82.98	3.12 (6.93*λ*)
4.0	18.71	0.365 (0.811*λ*)	1.91 (4.24*λ*)	97.91	3.65 (8.11*λ*)
5.0	19.03	0.358 (0.796*λ*)	1.92 (4.27*λ*)	102.06	4.36 (9.69*λ*)
6.0	17.90	0.361 (0.802*λ*)	2.14 (4.76*λ*)	106.11	4.76 (10.58*λ*)
7.0	20.44	0.355 (0.789*λ*)	2.15 (4.78*λ*)	123.79	6.70 (14.89*λ*)

**Table 2:** Characteristic parameters of PNJ at *T* = 25 °C.

*d* (μm)	*I*_max_ (a.u.)	*F* (μm)	*L* (μm)	*Q*	*f* (μm)
1.0	1.79	0.419 (0.931*λ*)	3.50 (7.78*λ*)	14.95	2.21 (4.91*λ*)
7.0	1.67	0.391 (0.869*λ*)	2.58 (5.73*λ*)	11.02	6.10 (13.56*λ*)

**Table 3:** Characteristic parameters of PNJ at *T* = 85 °C.

*d* (μm)	*I*_max_ (a.u.)	*F* (μm)	*L* (μm)	*Q*	*f* (μm)
1.0	12.98	0.347 (0.771*λ*)	2.40 (5.33*λ*)	89.78	1.98 (4.40*λ*)
7.0	11.61	0.388 (0.862*λ*)	2.91 (6.47*λ*)	87.01	6.80 (15.11*λ*)

## Data Availability

Data is contained within the article.
